# Recurrences and related characteristics of gastric cancer.

**DOI:** 10.1038/bjc.1996.468

**Published:** 1996-09

**Authors:** Y. Maehara, Y. Emi, H. Baba, Y. Adachi, K. Akazawa, Y. Ichiyoshi, K. Sugimachi

**Affiliations:** Department of Surgery II, Faculty of Medicine, Kyushu University, Fukuoka, Japan.

## Abstract

We analysed data on 1117 patients with gastric cancer who were treated by curative resection. Attention was focused on invasion and a recurrence of the cancer. Based on a univariate analysis, death following a recurrence and prognosis were related to age of the patients, size of the tumour, tumour location, tumour tissue differentiation, growth pattern, depth of invasion, lymphatic and vascular invasion and lymph node metastasis. In proportion to the growth potential, determined by the level of proliferating cell nuclear antigen (PCNA) labelling, the death related to a recurrence was increased and the prognosis was poorer. Multivariate analysis showed that the three factors of serosal invasion, PCNA labelling index and lymph node dissection were independent prognostic factors. When sites of recurrence were analysed regarding each depth of invasion, haematogenous recurrence, in particular in the liver, occurred even in cases of an early invasion and many types of recurrences, including peritoneal recurrence, were noted in patients with an advanced state of invasion.


British Journal of Cancer (1996) 74, 975-979
© 1996 Stockton Press All rights reserved 0007-0920/96 $12.00

### Recurrences and related characteristics of gastric cancer

Y Maehara[1], Y Emi[1], H Baba[1], Y Adachi[1], K Akazawa[2], Y Ichiyoshi[1] and K Sugimachi[1]

*Departments of [1]Surgery II and [2] Medical Informatics, Faculty of Medicine, Kyushu University, Fukuoka, Japan.*

**Summary** We analysed data on 1117 patients with gastric cancer who were treated by curative resection. Attention was focused on invasion and a recurrence of the cancer. Based on a univariate analysis, death following a recurrence and prognosis were related to age of the patients, size of the tumour, tumour location, tumour tissue differentiation, growth pattern, depth of invasion, lymphatic and vascular invasion and lymph node metastasis. In proportion to the growth potential, determined by the level of proliferating cell nuclear antigen (PCNA) labelling, the death related to a recurrence was increased and the prognosis was poorer. Multivariate analysis showed that the three factors of serosal invasion, PCNA labelling index and lymph node dissection were independent prognostic factors. When sites of recurrence were analysed regarding each depth of invasion, haematogenous recurrence, in particular in the liver, occurred even in cases of an early invasion and many types of recurrences, including peritoneal recurrence, were noted in patients with an advanced state of invasion.

**Keywords:** gastric cancer; depth of invasion; proliferating cell nuclear antigen

With emphasis on early diagnosis and detection of gastric cancer, prophylactic lymph node dissection and post-operative chemotherapy, the prognosis of patients with gastric cancer has improved (Maehara *et al.*, 1994, 1995*a*). However, recurrences are likely to take on a variety of forms and in different organs, even after curative resection of a gastric cancer (Baba *et al.*, 1989; Ichiyoshi *et al.*, 1990; Moriguchi *et al.*, 1992*a*). Recurrences were noted at each level of depth of tumour invasion and the rate of recurrences was increased in proportion to the degree of depth of invasion (Moriguchi *et al.*, 1992*b*). Risk factors for peritoneal recurrence were reported to be serosal invasion and Borrmann type IV and those for haematogenous recurrence were lymph node metastasis and vessel involvement (Moriguchi *et al.*, 1992*a*). However, there are few reports on patterns of recurrence with regard to each depth of invasion. When designing a post-operative follow-up system for gastric cancer after curative resection, it is important to examine the type of recurrence at each depth of tumour advance into the stomach wall. We determined clinicopathological and biological characteristics of gastric cancer with recurrences, and the type of recurrence at each level of tumour invasion was given attention.

### Patients and methods

### *Patients and surgical treatment*

From 1965 to 1987, 1117 Japanese patients with gastric cancer and no evidence of any other malignancy underwent gastric curative resection in the Department of Surgery II, Kyushu University Hospital, Fukuoka, Japan. Standardised procedures were: gastric resection was done, after determining the resection line 3 cm apart from the macroscopic edge for the localised tumour, and 6 cm for the infiltrative tumour (Kawasaki, 1975; Bozzetti *et al.*, 1982). Prophylactic lymph node dissection of more than D2 resection was carried out (Maehara *et al.*, 1992). Complete excision of invaded organs was done irrespective of the number of sites on the organs, when there was no evidence of incurable factors such as peritoneal dissemination, liver metastasis and widespread nodal involvement (Korenaga *et al.*, 1988*a*). All patients were examined clinically and pathologically with respect to the factors given in Table I. Pathological diagnosis and classification of the resected gastric cancer tissues were made according to the General Rules for Gastric Cancer Study in Surgery and Pathology in Japan (Japanese Research Society for Gastric Cancer, 1981, 1993). The lymph nodes in groups 1,2 and 3 are referred to as n1, n2 and n3, respectively, based on lymph node metastasis. Lymph node dissection was classified as follows: D1, complete removal of group 1 lymph node alone; D2, complete removal of group 1 and 2 lymph nodes; and D3, complete removal of group 1,2 and 3 lymph nodes.

Eight patients (0.7%) died within the first 30 post-operative days and outcome for two (0.2%) was unknown. Of the 1107 patients, 475 are alive, 351 died with a recurrence of the gastric cancer, 248 died with another disease and 33 died of undetermined causes. Death owing to causes other than gastric cancer were considered as censored data in the statistical analysis.

### *Staining for proliferating cell nuclear antigen (PCNA)*

Sections of 5 $\mu$m from paraffin blocks were dewaxed in xylene, rehydrated through a graded series of ethanol and immersed in 3% hydrogen peroxide in methanol. These sections were subsequently washed in phosphate-buffered saline, and normal goat serum was applied to reduce non-specific binding. The primary antibody PC10, a monoclonal mouse antibody for rat PCNA, was purchased from Dako Corp (Carpinteria, CA, USA). The sections were incubated for 2 h with PC10 (dilution 1:20) at room temperature, then with biotinylated goat anti-mouse IgG (1:200 for 1 h), and finally with the avidin-biotin-peroxidase complex (Kakeji *et al.*, 1994). Peroxidase labelling was developed with 3,3′-diaminobenzidine and hydrogen peroxide, and the sections were counterstained with haematoxylin. All stained nuclei were scored as positive for PCNA. The PCNA labelling index was determined by observing 1000 nuclei in areas of the section with the highest labelling frequency, and the percentage of PCNA-labelled nuclei (PCNA labelling index) was used for analysis.

### *Statistical analysis*

The BMDP statistical package program (BMDP; Los Angeles, CA, USA) for the IBM (Armonk, NY, USA) 3090 mainframe computer was used for all analyses (Dixon, 1988). The BMDP P4F and P3S programs were used for the chi-square test and the Kruskal-Wallis test to compare data on the groups. The BMDP P1L program was used to analyse survival time by the Kaplan-Meier method and the Mantel-Cox test was used to test for equality of the survival curves.

Correspondence: Y Maehara
Received 1 January 1996; revised 25 March 1996; accepted 28 March 1996

The BMDP P2L program was used for simultaneous multivariate adjustment of all covariates by the Cox regression analysis with forward stepwise model (Cox, 1972; Maehara *et al*., 1991*a*). The level of significance was $P<0.05$.

### Results

### *Clinicopathological factors*

At the time of analysis, the median follow-up time was 12.0 years for recurrence-free patients. Table I shows clinicopathological data on the 756 recurrence-free patients and on the 351 patients who died following a recurrence, all of whom underwent gastric resection. The rate of a recurrence-related death and 5 year survival was analysed regarding each clinicopathological factor. There was no gender difference in the rate of recurrence and prognosis. Death related to a recurrence was increased and the prognosis was poorer with increasing age of the patient and with tumour size. When the entire stomach was involved, the tumour was undifferentiated with infiltrative growth and the rates of lymphatic and vascular involvement and lymph node metastasis were higher, the rate of death related to recurrence was higher and the prognosis was poorer. In proportion to the depth of invasion, the recurrence-related death was increased and the prognosis was also poorer.

**Table I** Clinicopathological factors and recurrence of gastric cancer

| *Factor* | *Total* (n = *1107*) | *Recurrence-free* (n = *756*) | *Recurrence and death* (n = *351*) | *Five-year survival* (*%*) | P-*value*[a] |
|---|---|---|---|---|---|
| Sex | | | | | |
| Male | 760 (100.0) | 515 (67.8) | 245 (32.2) | 73.5 | 0.6648 |
| Female | 347 (100.0) | 241 (69.5) | 106 (30.5) | 73.8 | |
| Age (years) | | | | | |
| ⩽49 | 256 (100.0) | 186 (72.7) | 70 (27.3) | 77.9 | 0.0079 |
| 50 - 69 | 637 (100.0) | 430 (67.5) | 207 (32.5) | 73.9 | |
| ⩾70 | 214 (100.0) | 140 (65.4) | 74 (34.6) | 66.8 | |
| Maximum tumour diameter (cm) | | | | | |
| ⩽4.9 | 516 (100.0) | 440 (85.3) | 76 (14.7) | 89.8 | <0.0001 |
| 5.0 - 9.9 | 470 (100.0) | 276 (58.7) | 194 (41.3) | 63.9 | |
| ⩾10.0 | 121 (100.0) | 40 (33.1) | 81 (66.9) | 40.8 | |
| Tumour location | | | | | |
| Upper | 224 (100.0) | 134 (59.8) | 90 (40.2) | 64.7 | 0.0001 |
| Middle | 369 (100.0) | 282 (76.4) | 87 (23.6) | 82.5 | |
| Lower | 469 (100.0) | 325 (69.3) | 144 (30.7) | 74.8 | |
| Whole stomach | 45 (100.0) | 15 (33.3) | 30 (66.7) | 29.4 | |
| Histology | | | | | |
| Differentiated | 569 (100.0) | 414 (72.8) | 155 (27.2) | 78.1 | 0.0004 |
| Undifferentiated | 532 (100.0) | 337 (63.3) | 195 (36.7) | 68.8 | |
| Specific[b] | 4 | 3 | 1 | | |
| Unknown[b] | 2 | 2 | 0 | | |
| Depth of invasion | | | | | |
| m | 203 (100.0) | 192 (94.6) | 11 (5.4) | 96.8 | <0.0001 |
| sm | 213 (100.0) | 192 (90.1) | 21 (9.9) | 95.6 | |
| pm | 125 (100.0) | 90 (72.0) | 35 (28.0) | 81.1 | |
| ss | 130 (100.0) | 86 (66.2) | 44 (33.8) | 77.7 | |
| se | 380 (100.0) | 180 (47.4) | 200 (52.6) | 51.5 | |
| si and sei | 56 (100.0) | 16 (28.6) | 40 (71.4) | 27.1 | |
| Histological growth pattern | | | | | |
| Expansive | 214 (100.0) | 174 (81.3) | 40 (18.7) | 87.1 | <0.0001 |
| Intermediate | 325 (100.0) | 223 (68.6) | 102 (31.4) | 75.8 | |
| Infiltrative | 438 (100.0) | 237 (54.1) | 201 (45.9) | 59.5 | |
| Unknown[b] | 130 | 122 | 8 | | |
| Lymphatic involvement | | | | | |
| ly(−) | 494 (100.0) | 408 (82.6) | 86 (17.4) | 85.4 | <0.0001 |
| ly(+) | 357 (100.0) | 183 (51.3) | 174 (48.7) | 56.1 | |
| Unknown[b] | 256 | 165 | 91 | | |
| Vascular involvement | | | | | |
| v(−) | 693 (100.0) | 506 (73.0) | 187 (27.0) | 76.7 | <0.0001 |
| v(+) | 107 (100.0) | 54 (50.5) | 53 (49.5) | 53.6 | |
| Unknown[b] | 307 | 196 | 111 | | |
| Lymph node metastasis | | | | | |
| n(−) | 613 (100.0) | 522 (73.0) | 91 (14.8) | 91.0 | <0.0001 |
| n(+) | 494 (100.0) | 234 (47.4) | 260 (52.6) | 52.2 | |
| Gastric resection | | | | | |
| Partial | 759 (100.0) | 567 (74.7) | 192 (25.3) | 81.0 | <0.0001 |
| Total | 329 (100.0) | 177 (53.8) | 152 (46.2) | 55.9 | |
| Unknown[b] | 19 | 12 | 7 | | |
| Lymph node dissection | | | | | |
| D0 and D1 | 108 (100.0) | 74 (68.5) | 34 (31.5) | 76.1 | 0.9587 |
| D2 and D3 | 999 (100.0) | 682 (68.3) | 317 (31.7) | 73.4 | |

[a], Log-rank analysis. [b], Cases excluded from statistical analysis. m, mucosa; sm, submucosa; pm, muscularis propria; ss, subserosa; s.e, serosa (exposed); si, serosa (infiltrating adjacent tissue), sei, coexistance of exposed and infiltrating serosa. Percentages in brackets.

**Table II** PCNA labelling index and recurrence of gastric cancer

| *Factor* | *Total* (n=*155*) | *Recurrence-free* (n=91) | *Recurrence and death* (n=64) | *Five-year survival* (%) | P-*value* |
|---|---|---|---|---|---|
| PCNA labelling index (%) | | | | | |
| <20.0 | 47 (100.0) | 45 (95.7) | 2 (4.3) | 96.8 | <0.0001 |
| 20.0 - 39.9 | 55 (100.0) | 37 (67.3) | 18 (32.7) | 69.2 | |
| ⩾40 | 53 (100.0) | 9 (17.0) | 44 (83.0) | 20.0 | |

Percentages in brackets.

### *PCNA labelling*

Growth potential of gastric cancer tissues was evaluated by PCNA labelling and the level was grouped into three. The recurrence-related death was increased and the prognosis was poorer in cases of a higher PCNA labelling ($P<0.0001$) (Table II).

### *Multivariate analysis*

To determine which of the covariates listed in Table I and the PCNA labelling index were important prognostic factors of survival time for patients with gastric cancer, a multivariate analysis with Cox regression analysis with forward stepwise model was done for 155 cases. Serosal invasion, the PCNA labelling index and extended lymph node dissection proved to be independent factors ($P<0.05$) (Table III).

### *Recurrence pattern*

Data on 351 patients who died with a recurrence were analysed with respect to the pattern of recurrence (Table IV). Recurrences were detected at multiple sites in 40 patients. Haematogenous recurrences including liver recurrence were noted at each level of invasion. In cases of advanced invasion, a variety of areas of recurrence, including the peritoneum and distant organs of lung, bone and brain was evident. With regard to change in the rate of peritoneal recurrence at each depth of invasion, there was a significant difference among the six groups with regard to depth of invasion ($P=0.0251$). Local recurrences were equal at each level of invasion into the gastric wall. There was no difference in the rate of lymph node recurrence.

**Table III** Cox regression analysis for patients with gastric cancer

| *Prognostic factor (observed value)* | P-*value* | *Relative risk (95% confidence interval)* | |
|---|---|---|---|
| Serosal invasion (none, present) | <0.0001 | 6.5427 | (2.0513 - 20.810) |
| PCNA labelling index (per one labelling index) | <0.0001 | 1.1298 | (1.0874 - 1.1739) |
| Lymph node dissection (D0 and D1, D2 and D3) | <0.01 | 0.0905 | (0.0210 - 0.3886) |

### *Survival rates*

The survival rate for patients with gastric cancer was compared with each level of depth of invasion (Figure 1). Death from causes other than gastric cancer was considered as censored data in the survival analysis. Post-operative survival curve was poorer in proportion to the level of depth of invasion ($P<0.0001$).

### Discussion

The T factor of the TNM classification for gastric cancer was determined by the UICC organisation, according to depth of invasion into the gastric wall (Hermanek and Sobin, 1987). The type of recurrence varied with the characteristics of

**Figure 1** Survival curve as related to each depth of invasion following curative resection of gastric cancer. The survival time of patients with gastric cancer was shorter in proportion to the degree of depth of invasion. A, mucosa; B, submucosa; C, muscullaris propria; D, subserosa; E, serosa (exposed); F, serosa (infiltrating adjacent tissue) and coexistence of exposed and infiltrating serosa. ($P<0.0001$).

**Table IV** Sites of recurrence after resection of gastric cancer for each depth of invasion

| *Site of recurrence* | *Mucosa* (n=*11*) | *Submucosa* (n=*21*) | *Muscularis propia* (n=*35*) | *Subserosa* (n=*44*) | *Serosa (exposed)* (n=*200*) | *Serosa (infiltrating), exposed and infiltrating serosa* (n=*40*) |
|---|---|---|---|---|---|---|
| Peritoneum[a] | 2 (18.2) | 4 (19.0) | 7 (20.0) | 6 (13.6) | 72 (36.0) | 13 (32.5) |
| Haematogenous | 5 (45.5) | 8 (38.1) | 17 (48.6) | 21 (47.7) | 60 (30.0) | 18 (45.0) |
| Liver | 4 | 6 | 9 | 15 | 30 | 11 |
| Lung | 1 | 1 | 3 | 4 | 12 | 3 |
| Bone | 0 | 1 | 3 | 1 | 8 | 1 |
| Brain | 0 | 0 | 2 | 1 | 10 | 3 |
| Local | 2 (18.2) | 2 (9.5) | 5 (14.3) | 5 (11.4) | 31 (15.5) | 6 (15.0) |
| Lymph node | 0 | 0 | 1 (2.9) | 0 | 10 (5.0) | 3 (7.5) |
| Others, unknown | 4 (36.4) | 10 (47.6) | 11 (31.4) | 17 (38.6) | 54 (27.0) | 6 (15.0) |

Percentages in brackets. [a]$P=0.0251$, rate of peritoneal recurrence varied significantly among the six groups regarding depth of invasion.

gastric cancer and the incidence was increased in proportion to the depth of invasion. In the present work, age of the patient, tumour advances, including the depth of invasion and the growth potential of cancer cells and the extended lymph node dissection were closely related to the recurrence of the cancer and the prognosis (Maehara *et al.*, 1991*a*; Adachi *et al.*, 1994). When we analysed recurrences with regard to each depth of invasion, haematogenous recurrences, in particular liver recurrence, were apparent, irrespective of the depth of invasion. The rate of recurrences was lower in cases of early depth of invasion in which the tumours invaded mucosal and submucosal layers (Moreaux and Bougaran, 1993; Maehara *et al.*, 1993). Therefore, the incidence of haematogenous metastasis was relatively higher in cases of early gastric cancer (Ichiyoshi *et al.*, 1990; Orita *et al.*, 1992). Haematogenous recurrence was thought to occur mainly when cancer cells released from the primary site entered the vessel system and were transported to the organ, where attachment and proliferation occurred (Noguchi, 1990). The main locus of vascular invasion was seen most frequently in the submucosal layer (Noguchi, 1990), thus liver metastasis can occur even in the early stage of gastric cancer, in vascular invasion-positive cases. We reported the possible role of vascular and lymphatic spread of gastric cancer to distant organs in advanced stages of the disease and that vascular invasion and lymph node metastasis were independent risk factors of synchronous and metachronous liver metastasis (Maehara *et al.*, 1991*b*, 1995*b*; Moriguchi *et al.*, 1992*a*).

There are reports on the clinical significance of DNA ploidy, oncogene, growth factor and tumour marker for predicting the tumour progression and the survival of patients (Korenaga *et al.*, 1988*b*; Maehara *et al.*, 1990; Joypaul *et al.*, 1994; Lee *et al.*, 1994; Hirono *et al.*, 1995). We found that in proportion to the level of PCNA labelling of gastric cancer tissues, the recurrence rate was increased and the prognosis was poorer. PCNA is a highly conserved 36 kDa acidic protein and in conjunction with activator 1, acts as a processivity factor for DNA polymerase $\delta$ which is directly involved in DNA synthesis. The level of PCNA correlates with the proliferative state of cells determined by the Ki-67 score, bromodeoxyuridine incorporation and flow cytometry (Waseem and Lane, 1990; Hall *et al.*, 1990). As PCNA can be readily assayed using paraffin sections, the prognostic significance of the proliferating activity of cancer cells can be determined by the level of PCNA. The clinical usefulness of the PCNA level for predicting prognosis of patients with gastric cancer has been reported (Kakeji *et al.*, 1991; Maeda *et al.*, 1994). When the PCNA level was analysed by Cox regression analysis in these 155 cases, this factor proved to be prognostically significant. The growth potential of cancer cells is one approach to assess the likelihood of death from a recurrence of the gastric cancer.

The undifferentiated tissue with infiltrative growth was mainly noted in cases of an advanced level of invasion into the serosal layer or adjacent organs (Moriguchi *et al.*, 1992*b*). Peritoneal recurrence is dominant for an advanced stage of gastric cancer of the undifferentiated tissue type, serosal invasion and lymph node metastasis. In these cases, the tumour cells infiltrate the gastric wall and penetrate the serosa. Undifferentiated cancer cells are thought to disseminate transserosally to the peritoneum. Moreover, in cases of an advanced level of invasion, lymphatic and/or vascular routes account for spread of the cancer cells to the peritoneum and distant organs (Maehara *et al.*, 1991*b*, 1995*b*). With respect to lymph node dissection, extended lymph node dissection was not significant in the univariate analysis, however this factor was prognostically significant in the multivariate analysis, as reported previously (Maehara *et al.*, 1991*a*; Adachi *et al.*, 1994; Baba *et al.*, 1995). As a number of factors showed a close relation, a multivariate analysis is crucial for analysing the independent factors involved in the clinical outcome.

Our analysis of all the data shows that serosal invasion, growth potential and extended lymph node dissection are important prognostic factors and the type of recurrence varies at each level of invasion of the gastric tumour.

**Acknowledgements**
We thank M Ohara for comments. This work was supported by a Grant-in-Aid for Scientific Research (C)(06671286) from the Ministry of Education, Science and Culture in Japan and Yokoyama Foundation for Clinical Pharmacology.

### References

ADACHI Y, KAMAKURA T, MORI M, BABA H, MAEHARA Y AND SUGIMACHI K. (1994). Prognostic significance of the number of positive lymph nodes in gastric carcinoma. *Br. J. Surg.*, **81**, 414–416.

BABA H, KORENAGA D, OKAMURA T, SAITO A AND SUGIMACHI K. (1989). Prognostic factors in gastric cancer with serosal invasion. *Arch. Surg.*, **124**, 1061–1064.

BABA H, MAEHARA Y, TAKEUCHI H, INUTSUKA S, OKUYAMA T, ADACHI Y, AKAZAWA K AND SUGIMACHI K. (1995). Effect of lymph node dissection on the prognosis in patients with node-negative early gastric cancer. *Surgery*, **117**, 165–169.

BOZZETTI F, BONFANTI G, BUFALINO R, MENOTTI V, PERSANO S, ANDREOLA S, DOCI R AND GENNARI L. (1982). Adequacy of margins of resection in gastrectomy for cancer. *Ann. Surg.*, **196**, 685–690.

COX DR. (1972). Regression models and life tables. *J. R. Stat. Soc. (B)*, **34**, 187–220.

DIXON WJ (ed.). (1988). *BMDP Statistical Software*. pp. 229–718. University of California Press. Berkeley.

HALL PA, LEVISON DA, WOODS AL, YU CC-W, KELLOCK DB, WATKINS JA, BARNES DM, GILLETT CE, CAMPLEJOHN R, DOVER R, WASEEM NH AND LANE DP. (1990). Proliferating cell nuclear antigen (PCNA) immunolocalization in paraffin sections: an index of cell proliferation with evidence of deregulated expression in some neoplasms. *J. Pathol.*, **162**, 285–294.

HERMANEK P AND SOBIN LH (ed.). (1987). *TNM Classification of Malignant Tumours*. Fourth, fully revised version. pp. 43–46. Springer-Verlag: Berlin.

HIRONO Y, TSUGAWA K, FUSHIDA S, NINOMIYA I, YONEMURA Y, MIYAZAKI I, ENDOU Y, TANAKA M AND SASAKI T. (1995). Amplification of epidermal growth factor receptor gene and its relationship to survival in human gastric cancer. *Oncology*, **52**, 182–188.

ICHIYOSHI Y, TODA T, MINAMISONO Y, NAGASAKI S, YAKEISHI Y AND SUGIMACHI K. (1990). Recurrence in early gastric cancer. *Surgery*, **107**, 489–495.

JAPANESE RESEARCH SOCIETY FOR GASTRIC CANCER. (1981). The General Rules for the Gastric Cancer Study in Surgery and Pathology. Part I. Clinical Classification. *Jpn. J. Surg.*, **11**, 127–139. Part II. Histological classification of gastric cancer. *Jpn. J. Surg.*, **11**, 140–145.

JAPANESE RESEARCH SOCIETY FOR GASTRIC CANCER. (1993). *The General Rules for Gastric Cancer Study*, 12th edn. (in Japanese). Kanehara: Tokyo.

JOYPAUL BV, HOPWOOD D, NEWMAN EL, QURESHI S, GRANT A, OGSTON SA, LANE DP AND CUSCHIERI A. (1994). The prognostic significance of the accumulation of p53 tumour-suppressor gene protein in gastric adenocarcinoma. *Br. J. Cancer*, **69**, 943–946.

KAKEJI Y, MAEHARA Y, ADACHI Y, BABA H, MORI M, FURUSAWA M AND SUGIMACHI K. (1994). Proliferating activity as a prognostic factor in Borrmann type 4 gastric carcinoma. *Br. J. Cancer*, **69**, 749–753.

KAWASAKI S. (1975). A clinicopathological study on upward intramural extension of cancer of the stomach. (in Japanese with English abstract). *Fukuoka Acta Medica*, **66**, 1–23.

KORENAGA D, OKAMURA T, BABA H, SAITO A. AND SUGIMACHI K. (1988*a*). Results of resection of gastric cancer extending to adjacent organs. *Br. J. Surg.*, **75**, 12–15.

KORENAGA D, OKAMURA T, SAITO A, BABA H AND SUGIMACHI K. (1988*b*). DNA ploidy is closely linked to tumor invasion, lymph node metastasis, and prognosis in clinical gastric cancer. *Cancer*, **62**, 309–313.

LEE EY, CIBULL ML, STRODEL WE AND HALEY JV. (1994). Expression of HER-2/neu oncoprotein and epidermal growth factor receptor and prognosis in gastric carcinoma. *Arch. Pathol. Lab. Med.*, **118**, 235–239.

MAEDA K, CHUNG YS, ONODA N, KATO Y, NITTA A, ARIMOTO Y, YAMADA N, KONDO Y AND SOWA M. (1994). Proliferating cell nuclear antigen labeling index of preoperative biopsy specimens in gastric carcinoma with special reference to prognosis. *Cancer*, **73**, 528–533.

MAEHARA Y, SUGIMACHI K, AKAGI M, KAKEGAWA T, SHIMAZU H AND TOMITA M. (1990). Serum carcinoembryonic antigen level increases correlation with tumor progression in patients with differentiated gastric carcinoma following noncurative resection. *Cancer Res.*, **50**, 3952–3955.

MAEHARA Y, MORIGUCHI S, YOSHIDA M, TAKAHASHI I, KORENAGA D AND SUGIMACHI K. (1991*a*). Splenectomy does not correlate with length of survival in patients undergoing curative total gastrectomy for gastric carcinoma. *Cancer*, **67**, 3006–3009.

MAEHARA Y, MORIGUCHI S, KAKEJI Y, KOHNOE S, KORENAGA D, HARAGUCHI M AND SUGIMACHI K. (1991*b*). Pertinent risk factors and gastric carcinoma with synchronous peritoneal dissemination or liver metastasis. *Surgery*, **110**, 820–823.

MAEHARA Y, OKUYAMA T, MORIGUCHI S, ORITA H, KUSUMOTO H, KORENAGA D AND SUGIMACHI K. (1992). Prophylactic lymph node dissection in patients with advanced gastric cancer promotes increased survival time. *Cancer*, **70**, 392–395.

MAEHARA Y, OKUYAMA T, OSHIRO T, BABA H, ANAI H, AKAZAWA K AND SUGIMACHI K. (1993). Early carcinoma of the stomach. *Surg. Gynecol. Obstet.*, **177**, 593–597.

MAEHARA Y, OKUYAMA T, KAKEJI Y, BABA H, FURUSAWA M AND SUGIMACHI K. (1994). Postoperative immunochemotherapy including Streptococcal lysate OK-432 is effective for patients with gastric cancer and serosal invasion. *Am. J. Surg.*, **168**, 36–40.

MAEHARA Y, OSHIRO T, OIWA H, ODA S, BABA H, AKAZAWA K AND SUGIMACHI K. (1995*a*). Gastric cancer in patients over 70 years of age. *Br. J. Surg.*, **82**, 102–105.

MAEHARA Y, OSHIRO T, BABA H, OHNO S, KOHNOE S AND SUGIMACHI K. (1995*b*). Lymphatic invasion and potential for tumor growth and metastasis in patients with gastric cancer. *Surgery*, **117**, 380–385.

MOREAUX J AND BOUGARAN J. (1993). Early gastric cancer. A 25-year surgical experience. *Ann. Surg.*, **217**, 347–355.

MORIGUCHI S, MAEHARA Y, KORENAGA D, SUGIMACHI K AND NOSE Y. (1992*a*). Risk factors which predict pattern of recurrence after curative surgery for patients with advanced gastric cancer. *Surg. Oncol.*, **1**, 341–346.

MORIGUCHI S, MAEHARA Y, KORENAGA D, KAKEJI Y, SUGIMACHI K AND NOSE Y. (1992*b*). The relationship between prognostic significance of pathological type and the degree of gastric wall invasion in gastric cancer. *Cancer J.*, **5**, 220–223.

NOGUCHI Y. (1990). Blood vessel invasion in gastric carcinoma. *Surgery*, **107**, 140–148.

ORITA H, MATSUSAKA T, WAKASUGI K, KUME K, FUJINAGA Y, FUCHIGAMI T AND IWASHITA A. (1992). Clinicopathologic evaluation of recurrence in early gastric cancer. *Surgery Today*, **22**, 19–23.

WASEEM NH AND LANE DP. (1990). Monoclonal antibody analysis of the proliferating cell nuclear antigen (PCNA). Structural conservation and the detection of a nucleolar form. *J. Cell Sci.*, **96**, 121–129.


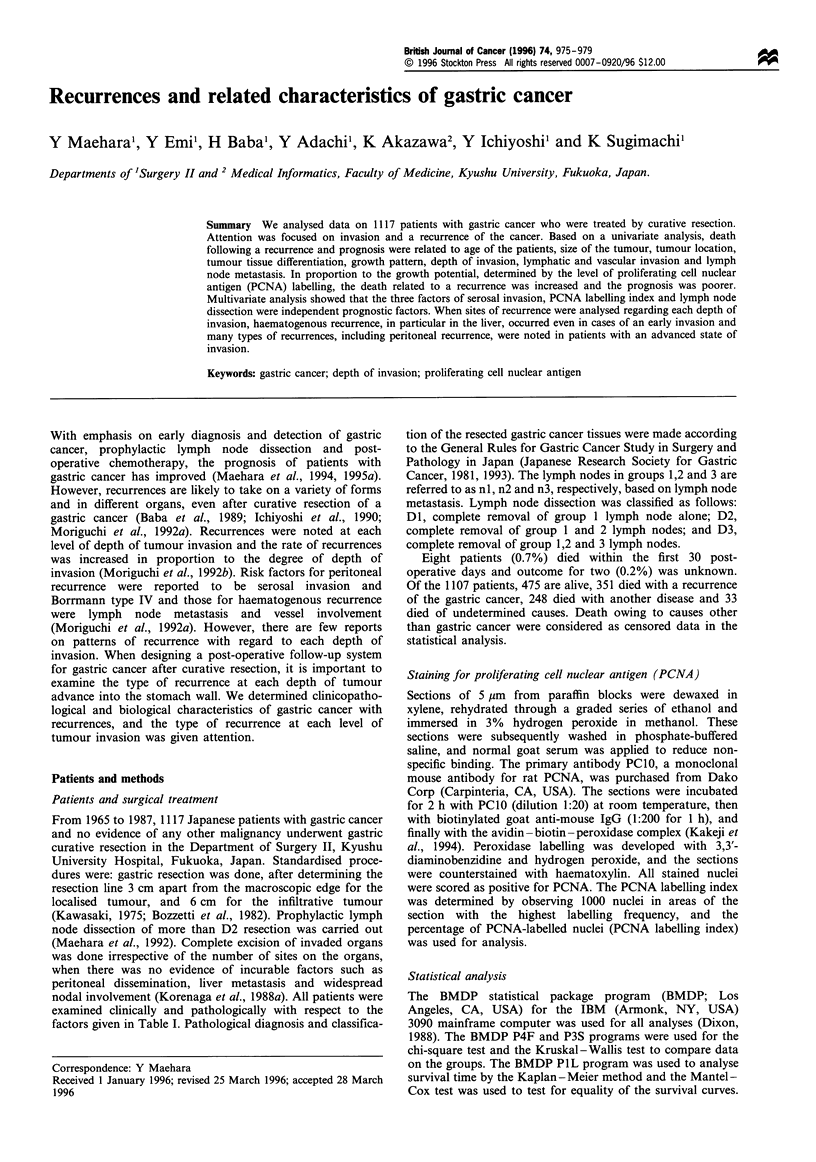

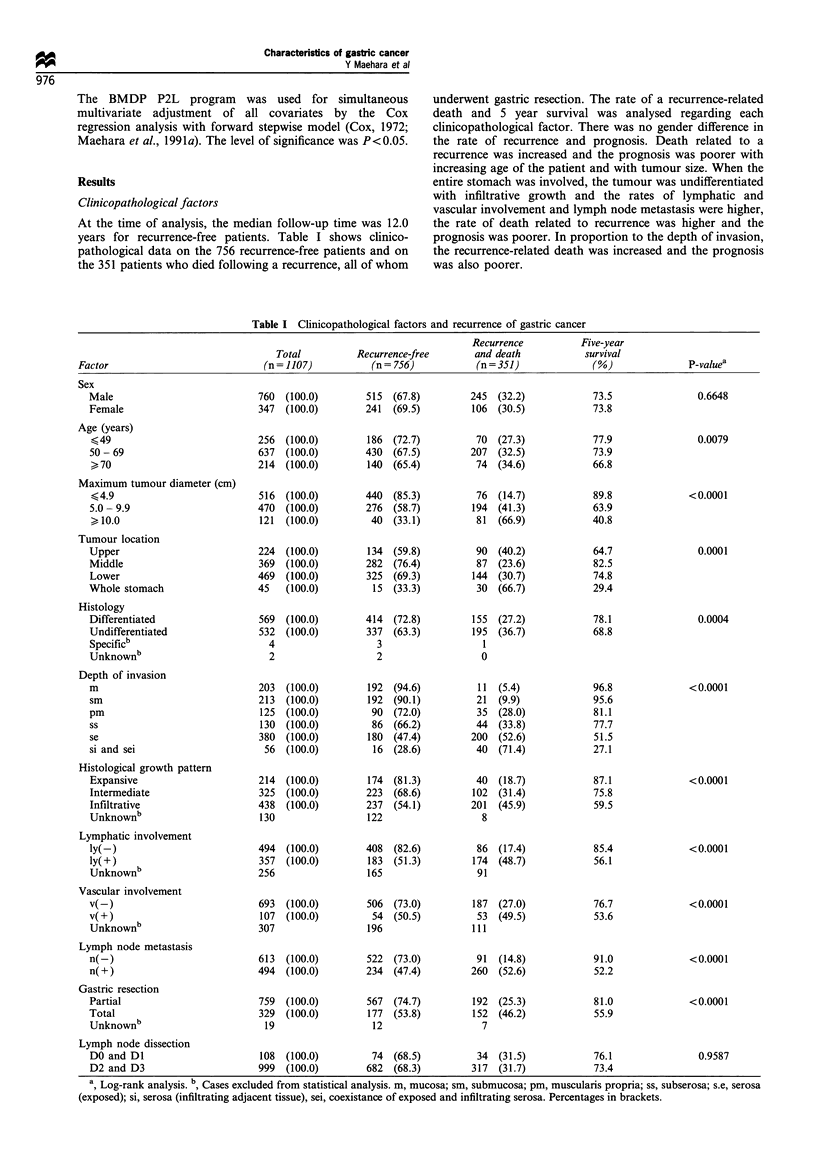

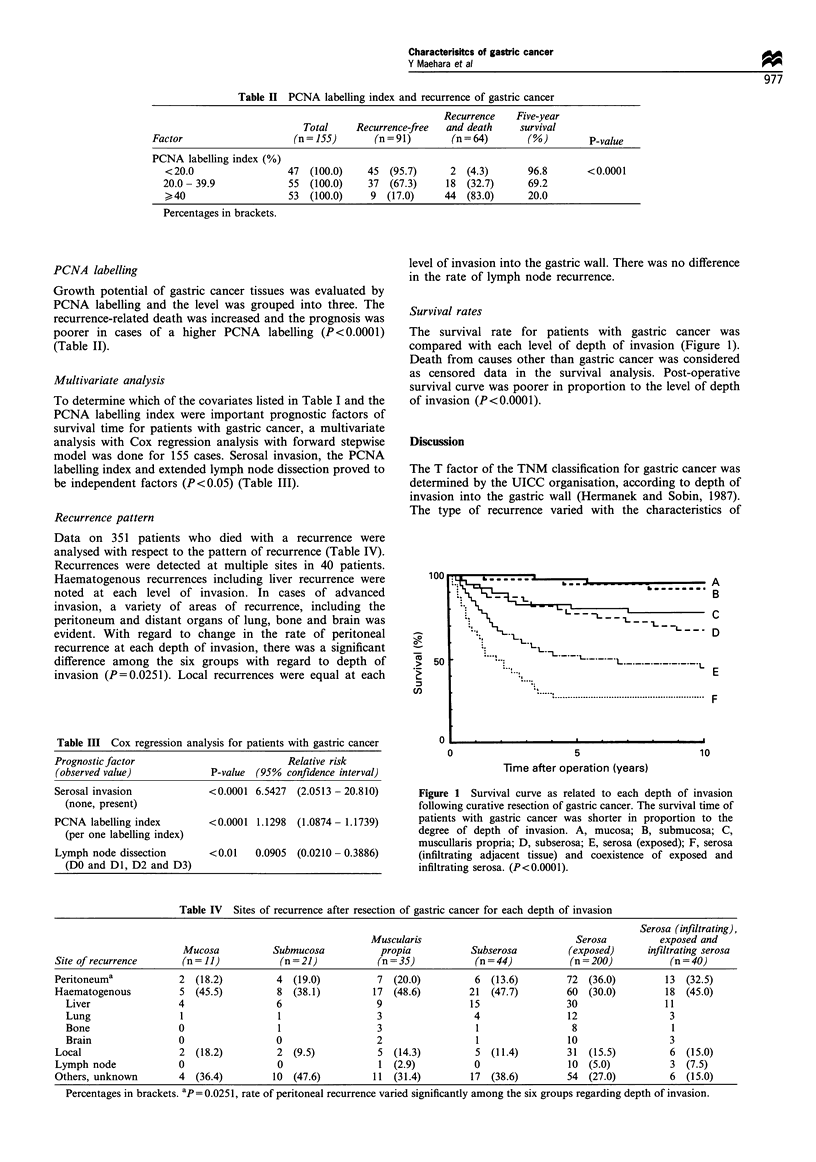

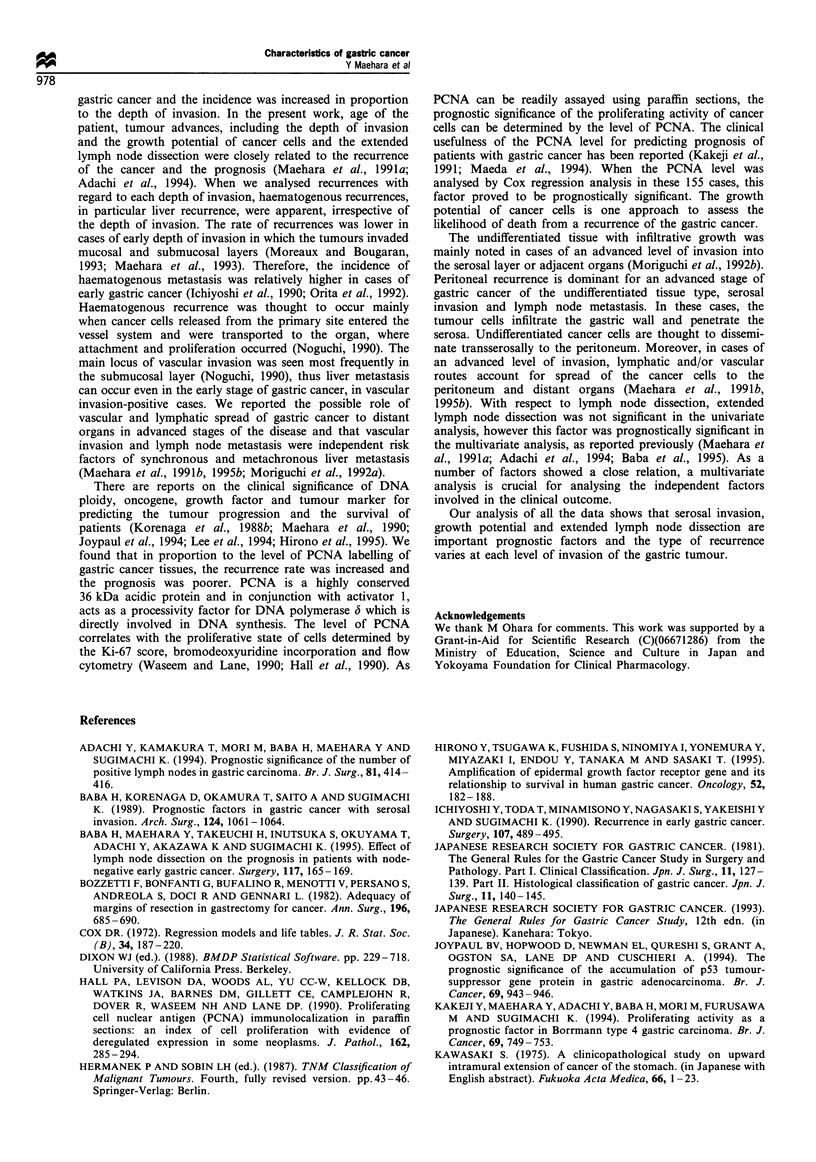

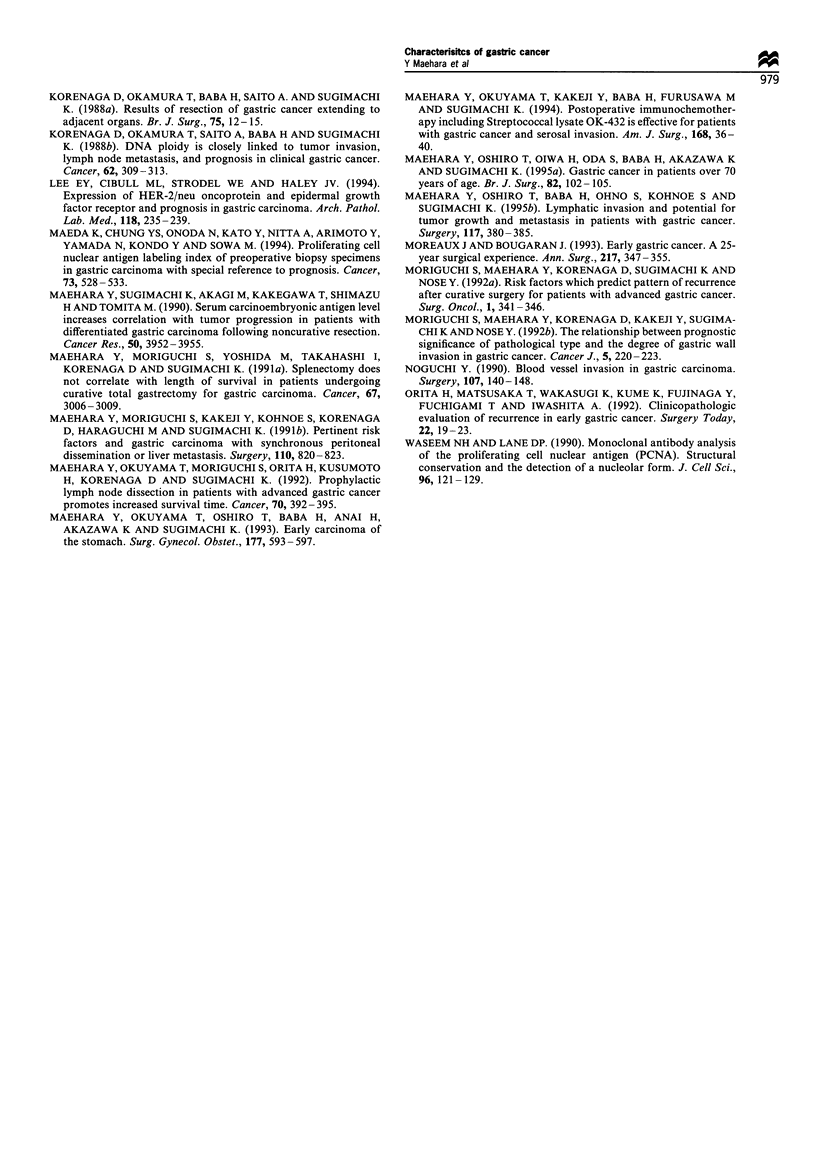


## References

[OCR_00815] Adachi Y., Kamakura T., Mori M., Baba H., Maehara Y., Sugimachi K. (1994). Prognostic significance of the number of positive lymph nodes in gastric carcinoma.. Br J Surg.

[OCR_00820] Baba H., Korenaga D., Okamura T., Saito A., Sugimachi K. (1989). Prognostic factors in gastric cancer with serosal invasion. Univariate and multivariate analyses.. Arch Surg.

[OCR_00823] Baba H., Maehara Y., Takeuchi H., Inutsuka S., Okuyama T., Adachi Y., Akazawa K., Sugimachi K. (1995). Effect of lymph node dissection on the prognosis in patients with node-negative early gastric cancer.. Surgery.

[OCR_00829] Bozzetti F., Bonfanti G., Bufalino R., Menotti V., Persano S., Andreola S., Doci R., Gennari L. (1982). Adequacy of margins of resection in gastrectomy for cancer.. Ann Surg.

[OCR_00843] Hall P. A., Levison D. A., Woods A. L., Yu C. C., Kellock D. B., Watkins J. A., Barnes D. M., Gillett C. E., Camplejohn R., Dover R. (1990). Proliferating cell nuclear antigen (PCNA) immunolocalization in paraffin sections: an index of cell proliferation with evidence of deregulated expression in some neoplasms.. J Pathol.

[OCR_00860] Hirono Y., Tsugawa K., Fushida S., Ninomiya I., Yonemura Y., Miyazaki I., Endou Y., Tanaka M., Sasaki T. (1995). Amplification of epidermal growth factor receptor gene and its relationship to survival in human gastric cancer.. Oncology.

[OCR_00867] Ichiyoshi Y., Toda T., Minamisono Y., Nagasaki S., Yakeishi Y., Sugimachi K. (1990). Recurrence in early gastric cancer.. Surgery.

[OCR_00881] Joypaul B. V., Hopwood D., Newman E. L., Qureshi S., Grant A., Ogston S. A., Lane D. P., Cuschieri A. (1994). The prognostic significance of the accumulation of p53 tumour-suppressor gene protein in gastric adenocarcinoma.. Br J Cancer.

[OCR_00888] Kakeji Y., Maehara Y., Adachi Y., Baba H., Mori M., Furusawa M., Sugimachi K. (1994). Proliferative activity as a prognostic factor in Borrmann type 4 gastric carcinoma.. Br J Cancer.

[OCR_00894] Kawasaki S. (1975). [A clinicopathological study on upward intramural extension of cancer of the stomach (author's transl)].. Fukuoka Igaku Zasshi.

[OCR_00907] Korenaga D., Okamura T., Baba H., Saito A., Sugimachi K. (1988). Results of resection of gastric cancer extending to adjacent organs.. Br J Surg.

[OCR_00912] Korenaga D., Okamura T., Saito A., Baba H., Sugimachi K. (1988). DNA ploidy is closely linked to tumor invasion, lymph node metastasis, and prognosis in clinical gastric cancer.. Cancer.

[OCR_00918] Lee E. Y., Cibull M. L., Strodel W. E., Haley J. V. (1994). Expression of HER-2/neu oncoprotein and epidermal growth factor receptor and prognosis in gastric carcinoma.. Arch Pathol Lab Med.

[OCR_00922] Maeda K., Chung Y. S., Onoda N., Kato Y., Nitta A., Arimoto Y., Yamada N., Kondo Y., Sowa M. (1994). Proliferating cell nuclear antigen labeling index of preoperative biopsy specimens in gastric carcinoma with special reference to prognosis.. Cancer.

[OCR_00946] Maehara Y., Moriguchi S., Kakeji Y., Kohnoe S., Korenaga D., Haraguchi M., Sugimachi K. (1991). Pertinent risk factors and gastric carcinoma with synchronous peritoneal dissemination or liver metastasis.. Surgery.

[OCR_00939] Maehara Y., Moriguchi S., Yoshida M., Takahashi I., Korenaga D., Sugimachi K. (1991). Splenectomy does not correlate with length of survival in patients undergoing curative total gastrectomy for gastric carcinoma. Univariate and multivariate analyses.. Cancer.

[OCR_00962] Maehara Y., Okuyama T., Kakeji Y., Baba H., Furusawa M., Sugimachi K. (1994). Postoperative immunochemotherapy including streptococcal lysate OK-432 is effective for patients with gastric cancer and serosal invasion.. Am J Surg.

[OCR_00951] Maehara Y., Okuyama T., Moriguchi S., Orita H., Kusumoto H., Korenaga D., Sugimachi K. (1992). Prophylactic lymph node dissection in patients with advanced gastric cancer promotes increased survival time.. Cancer.

[OCR_00958] Maehara Y., Okuyama T., Oshiro T., Baba H., Anai H., Akazawa K., Sugimachi K. (1993). Early carcinoma of the stomach.. Surg Gynecol Obstet.

[OCR_00972] Maehara Y., Oshiro T., Baba H., Ohno S., Kohnoe S., Sugimachi K. (1995). Lymphatic invasion and potential for tumor growth and metastasis in patients with gastric cancer.. Surgery.

[OCR_00970] Maehara Y., Oshiro T., Oiwa H., Oda S., Baba H., Akazawa K., Sugimachi K. (1995). Gastric carcinoma in patients over 70 years of age.. Br J Surg.

[OCR_00929] Maehara Y., Sugimachi K., Akagi M., Kakegawa T., Shimazu H., Tomita M. (1990). Serum carcinoembryonic antigen level increases correlate with tumor progression in patients with differentiated gastric carcinoma following noncurative resection.. Cancer Res.

[OCR_00978] Moreaux J., Bougaran J. (1993). Early gastric cancer. A 25-year surgical experience.. Ann Surg.

[OCR_00982] Moriguchi S., Maehara Y., Korenaga D., Sugimachi K., Nose Y. (1992). Risk factors which predict pattern of recurrence after curative surgery for patients with advanced gastric cancer.. Surg Oncol.

[OCR_00994] Noguchi Y. (1990). Blood vessel invasion in gastric carcinoma.. Surgery.

[OCR_00998] Orita H., Matsusaka T., Wakasugi K., Kume K., Fujinaga Y., Fuchigami T., Iwashita A. (1992). Clinicopathologic evaluation of recurrence in early gastric cancer.. Surg Today.

[OCR_01004] Waseem N. H., Lane D. P. (1990). Monoclonal antibody analysis of the proliferating cell nuclear antigen (PCNA). Structural conservation and the detection of a nucleolar form.. J Cell Sci.

